# The complete chloroplast genome sequence of *Lilium saccatum* S. Yun Liang (Liliaceae, Lilieae) and its phylogenetic analysis

**DOI:** 10.1080/23802359.2024.2403410

**Published:** 2024-09-16

**Authors:** Xuan Zhou, Jian-Hua Ma, Jun-Yi Zhang, Li Luan, Lin Li, Yun-Dong Gao, Yu Feng

**Affiliations:** a Yajiang Clean Energy Ecological and Environment (Chengdu) Co., Ltd., Chengdu, China; b PowerChina Chengdu Engineering Corporation Limited, Chengdu, China; c CAS Key Laboratory of Mountain Ecological Restoration and Bioresource Utilization & Ecological Restoration and Biodiversity Conservation Key Laboratory of Sichuan Province, Chengdu Institute of Biology, Chinese Academy of Sciences, Chengdu, China

**Keywords:** Chloroplast genome, *Lilium saccatum*, phylogeny

## Abstract

*Lilium saccatum* is a species of ornamental plant found in southeastern Xizang, China. In the present study, the complete chloroplast (cp) genome of *L. saccatum* was sequenced using next-generation sequencing (NGS). The *de novo* assembled cp genome was 151,839 bp in length, including a pair of inverted repeat regions (IRs; 26,421 bp), a small single-copy region (SSC; 17,528 bp), and a large single-copy region (LSC; 81,469 bp). The cp genome encodes 113 unique genes, including 79 protein-coding genes (PCGs), 30 tRNA genes, and four rRNA genes. The total GC content of the cp genome was 37.0%. Phylogenetic analysis of 24 cp genomes revealed that *L. saccatum* was closely related to *L. souliei*. This study could provide fundamental information for the phylogenomics and utilization of *Lilium*.

## Introduction

*Lilium saccatum* S.Yun Liang 1987 is a herbaceous plant distributed in the southeastern Xizang Province, China (Liang 1987). The purple flowers of *L. saccatum* typically have bell-shaped, and the ovate or elliptic-lanceolate leaves are scattered, occasionally several in dense and subwhorled clusters (Liang and Minoru 2000). Lilies are popular ornamentals in gardens and landscapes for their beautiful flowers and evergreen foliage. With the rapid development of next-generation sequencing (NGS) technology, it is becoming easier to extract the information of organelle genomes. The chloroplast (cp) genome can provide valuable information for species identification, genetics, evolution, and phylogeny owing to its conserved genome structure and high substitution rates compared to other organelles of the plant (Daniell et al. 2021; Li et al. 2021; Zhang et al. 2022). To better understand the taxonomic and evolutionary relationships of *Lilium*, we assembled the complete cp genome of *L. saccatum* based on Illumina pair-end sequencing data (San Diego, CA).

## Materials and methods

**Figure 1.**
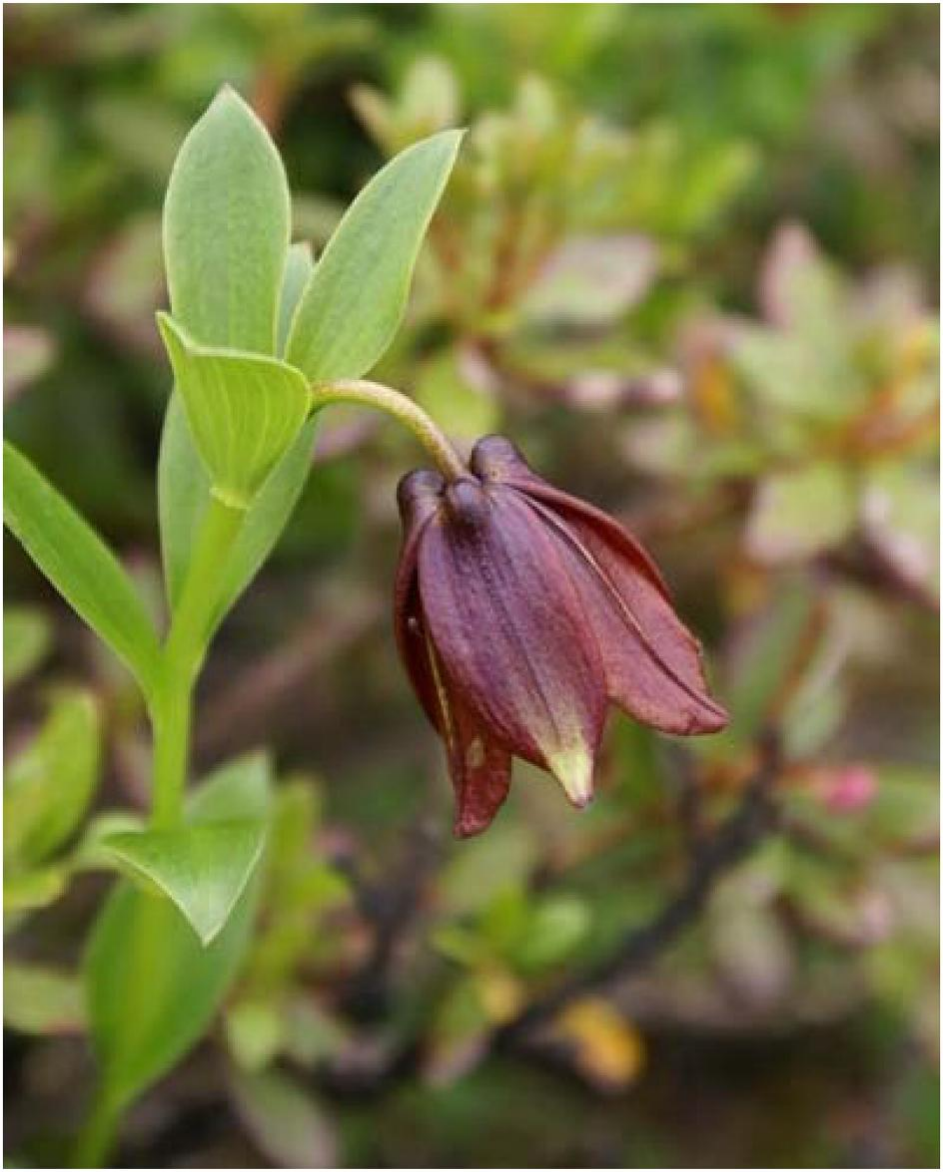
Photograph of *Lilium saccatum* (this unpublished photo, taken in Bomi County, Xizang Province, China by Mr. Shang-Hua Xia, is used with permission). Flower solitary, nodding and campanulate, tepals purple-red with dark spots adaxially.

The samples of *Lilium saccatum* (Figure 1) were grown in the mountain slope regions of the southeastern Xizang Province, China (Milin County: 29°12′29.47″ N, 94°09′50.31″ E). Voucher specimens and fresh leaves of *Lilium saccatum* (voucher: GYD-1406; contact person: Bo Xu, xubo@cib.ac.cn) were deposited at the Herbarium of Chengdu Institute of Biology (CDBI). Total genomic DNA was extracted from silica-gel dried leaves through Plant DNA Isolation Kit (Cat. No. DE-06111, Foregene, Chengdu, China) and sequenced via Illumina paired-end technology (San Diego, CA). *De novo* assembly of the cp genome was carried out using GetOrganelle v1.7.2 (Jin et al. 2020), and the average coverage for the assembled cp genome was 3645.04× (Figure S1). The assembled cp genome was annotated using PGA (Qu et al. 2019) and manually corrected for the start and stop codons. The final genome map of *L. saccatum* was generated using CPGview (http://www.1kmpg.cn/cpgview). The phylogenetic analysis was constructed based on 24 complete cp genomes, including 23 species of *Lilium* and *Fritillaria karelinii* (Fisch.) Baker 1874 as outgroup species. Sequences were aligned via MAFFT v7.475 (Katoh and Standley 2013). A maximum-likelihood (ML) method for phylogenetic analysis was performed via IQ-Tree v.2.1.4 (Nguyen et al. 2015) with 2000 ultrafast bootstrap replicates. The resulted phylogenetic trees were visualized using FigTree v1.4.4 (http://tree.bio.ed.ac.uk/software/figtree). We also compared the singleton variable sites between *L. saccatum* and *L. souliei* (Franch.) Sealy 1950 by using DnaSP v6.12.03 (Rozas et al. 2017).

## Results

**Figure 2.**
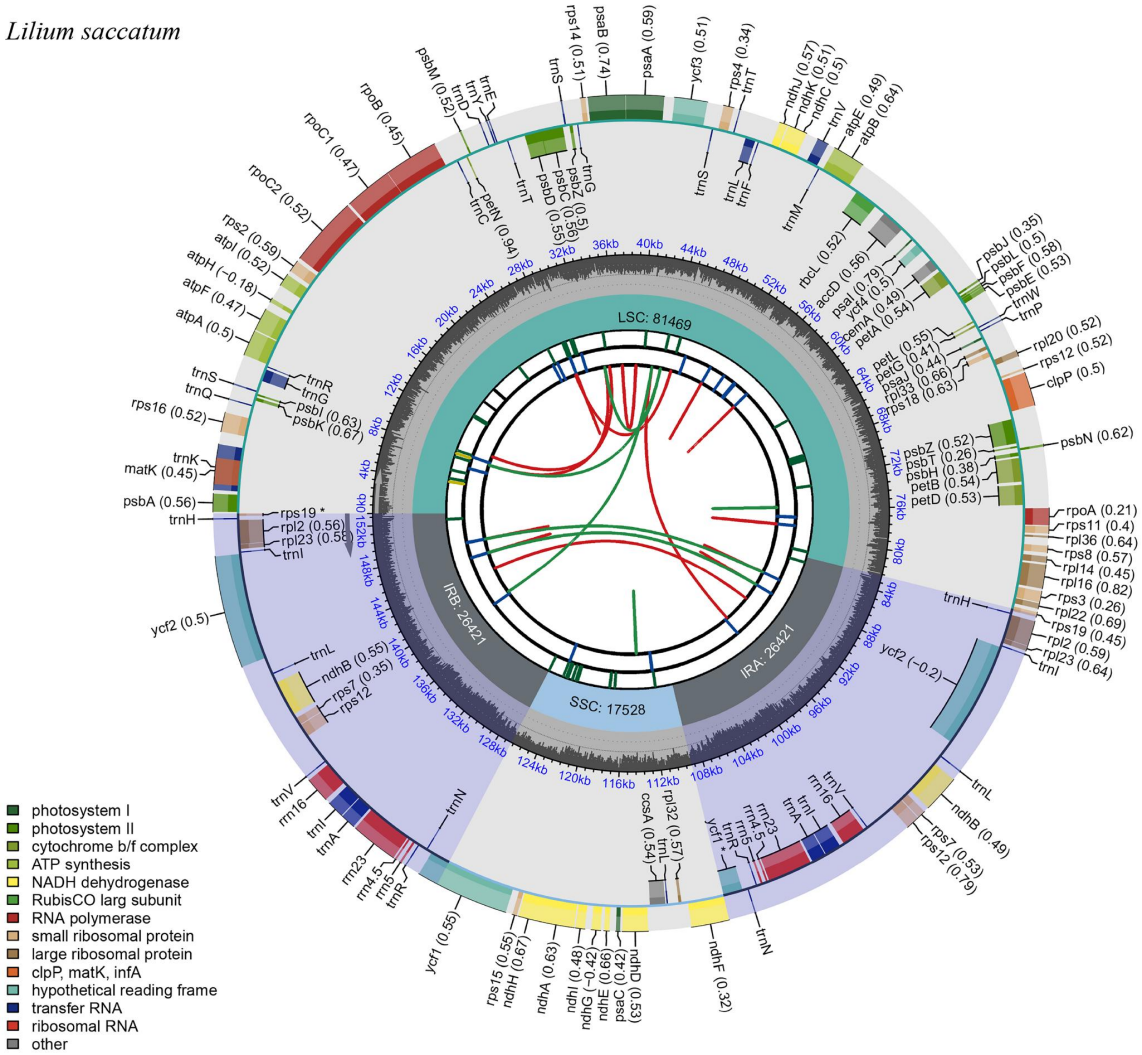
Chloroplast genome map of *Lilium saccatum*. The map was generated by CPGView. Genes located on the inner and outer of circle are transcribed clockwise and anticlockwise, respectively. The dark grey inner circle indicates GC content. Large single-copy (LSC), small single-copy (SSC), and inverted repeats (IRA and IRB) are indicated in the inner layer. The functional classification of the genes is provided in the bottom left corner.

**Figure 3.**
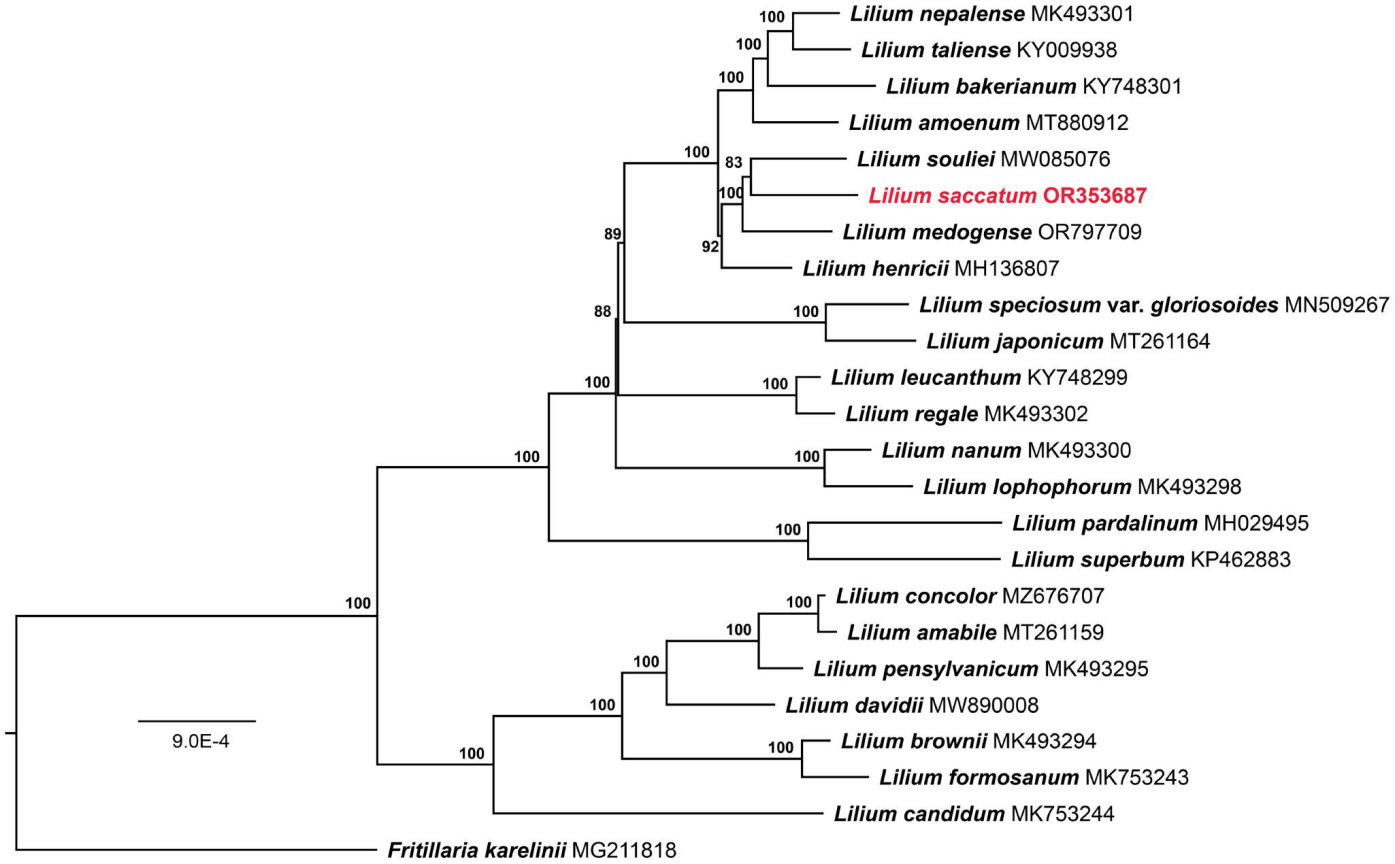
The maximum-likelihood phylogeny obtained from 24 complete chloroplast sequences. The accession numbers of used sequences follow the species names, and the newly sequenced genome was shown in red font. The 23 species were *Fritillaria karelinii* (MG211818, outgroup) (Li et al. 2017), *Lilium candidum* (MK753244) (Lu et al. 2021), *L. brownii* (MK493294) (Du et al. 2017), *L. formosanum* (MK753244) (Lu et al. 2021), *L. davidii* (MW890008) (Li et al. 2021), *L. pensylvanicum* (MK493295) (Du et al. 2017), *L. amabile* (MT261159) (Do et al. 2020), *L. concolor* (MZ676707) (Du et al. 2017), *L. pardalinum* (MH029495) (Kim et al. 2018), *L. superbum* (KP462883) (Mennes et al. 2015), *L. lophophorum* (MK493298) (Du et al. 2017), *L. nanum* (MK493300) (Du et al. 2017), *L. leucanthum* (KY748299) (Du et al. 2017), *L. nanum* (MK493303) (Du et al. 2017), *L. japonicum* (MT261164) (Do et al. 2020), *L. speciosum* var. *gloriosoides* (MN509267) (Liu et al. 2019), *L. henricii* (MH029495) (Kim et al. 2018), *L. medogense* (OR797709) (Yuan and Gao 2024), *L. saccatum* (OR353687, this study), *L. souliei* (MW085076) (Li et al. 2021), *L. amoenum* (MT880912) (Do et al. 2020), *L. bakerianum* (KY748301) (Du et al. 2017), *L. nepalense* (MK493301) (Du et al. 2017), and *L. taliense* (KY009938) (Du et al. 2017).

The cp genomes of *Lilium saccatum* and their sequenced raw data (GenBank number: OR353687; BioProject, SRA, and Bio-Sample numbers: PRJCA022586, CRR1005868, and SAMC3300505, respectively) were deposited in the NCBI (https://www.ncbi.nlm.nih.gov/) and CNCB (https://www.cncb.ac.cn/) database, respectively. The cp genome of *L. saccatum* was 151,839 bp in length; the quality control and read coverage depth map of the assembly of the cp genome are shown in Figure 2 and Figure S1. The overall GC content was 37.0%, which is higher than either large single-copy (LSC) regions (34.8%) or small single-copy (SSC) (30.6%) region, but lower than the inverted repeat (IR) (42.5%) region. It encodes 113 unique genes, including 79 protein-coding genes (PCGs), 30 tRNAs, and four rRNAs. Introns were detected in 20 genes, where 16 genes (*atpF*, *ndhA*, *ndhB*, *petB*, *petD*, *rpl16*, *rpl2*, *rpoC1*, *rps12*, *rps16*, *trnA-UGC*, *trnG-UCC*, *trnI-GAU*, *trnK-UUU*, *trnL-UAA*, and *trnV-UAC*) had a single intron, and two genes (*clpP1* and *pafI*) had two introns (Figure S2). The trans-splicing gene *rps12* had three unique exons (Figure S3). To clarify the system position of *L. saccatum*, we utilized a closely related genus species, *Fritillaria karelinii*, as outgroup and constructed ML tree based on 23 representative lilies, and the results showed that *L. saccatum* was closely clustered with *L. souliei* (Figure 3). Moreover, the aligned matrix of *L. saccatum* and *L. souliei* was 153,383 nucleotides in length with 565 singleton variable sites.

## Discussion and conclusions

In this study, we first reported the complete cp genome of *Lilium saccatum*, which was 151,839 bp in total length and had a typical quadripartite structure. A total of 113 unique genes were annotated in this plastome, which are not significantly different from other of published cp genomes in *Lilium* (Kim et al. 2017; Duan et al. 2022). The phylogeny reconstructed based on 23 complete cp genomes of *Lilium* reinforced the monophyly of this genus as a whole, which is consistent with previous studies (Du et al. 2017; Duan et al. 2022). Previous phylogenetic studies of the genus *Lilium* clarified *L. saccatum* was closely related to *L. souliei* based on cp gene sequences (Gao et al. 2013), and our phylogenetic results also reinforce this relationship. With the increase of sequenced species, the phylogenetic relationship on the genus *Lilium* will be clear. The complete cp genome of *L. saccatum* reported in this study is the first genomic resource for this species, a valuable resource for unraveling the evolutionary history of these high ornamental plants. Furthermore, our results provide a valuable resource for distribution, utilization, genetics, and phylogenetic studies of lilies.

## Data Availability

The data that support the findings of this study are openly available in GenBank number OR353687 (https://www.ncbi.nlm.nih.gov/nuccore/0R353687) and the related BioProject, raw sequencing files in SRA, and the Bio-Sample number are PRJCA022586, CRR1005868, and SAMC3300505 (https://www.cncb.ac.cn/services), respectively.
